# Long-Term Inhalation Exposure to Nickel Nanoparticles Exacerbated Atherosclerosis in a Susceptible Mouse Model

**DOI:** 10.1289/ehp.1002508

**Published:** 2010-09-23

**Authors:** Gi Soo Kang, Patricia Anne Gillespie, Albert Gunnison, Andre Luis Moreira, Kam-Meng Tchou-Wong, Lung-Chi Chen

**Affiliations:** 1 Department of Environmental Medicine, New York University School of Medicine, Tuxedo, New York, USA; 2 Department of Pathology, Memorial Sloan Kettering Cancer Center, New York, New York, USA

**Keywords:** atherosclerosis, cardiovascular toxicity, inhalation, nanoparticles, nickel

## Abstract

**Background:**

Because associations have been reported between inhaled ambient ultrafine particles and increased risk of cardiopulmonary disease, it has been suggested that inhaled engineered nanoparticles (NPs) may also induce adverse effects on the cardiovascular system.

**Objective:**

We examined the long-term cardiovascular effects of inhaled nickel hydroxide NPs (nano-NH) using a sensitive mouse model.

**Methods:**

Hyperlipidemic, apoprotein E-deficient (ApoE^−/−^) mice were exposed to nano-NH at either 0 or 79 μg Ni/m^3^, via a whole-body inhalation system, for 5 hr/day, 5 days/week, for either 1 week or 5 months. We measured various indicators of oxidative stress and inflammation in the lung and cardiovascular tissue, and we determined plaque formation on the ascending aorta.

**Results:**

Inhaled nano-NH induced significant oxidative stress and inflammation in the pulmonary and extrapulmonary organs, indicated by up-regulated mRNA levels of certain antioxidant enzyme and inflammatory cytokine genes; increased mitochondrial DNA damage in the aorta; significant signs of inflammation in bronchoalveolar lavage fluid; changes in lung histopathology; and induction of acute-phase response. In addition, after 5-month exposures, nano-NH exacerbated the progression of atherosclerosis in ApoE^−/−^ mice.

**Conclusions:**

This is the first study to report long-term cardiovascular toxicity of an inhaled nanomaterial. Our results clearly demonstrate that long-term exposure to inhaled nano-NH can induce oxidative stress and inflammation, not only in the lung but also in the cardiovascular system, and that this stress and inflammation can ultimately contribute to progression of atherosclerosis in ApoE^−/−^ mice.

Nanoparticles (NPs) are known for their unique characteristics, including small size, large surface area, and high reactivity. Although these characteristics make NPs attractive for various medical and technological applications, they may also affect the potential toxicity of NPs. Indeed, various studies have shown that, on an equal-mass basis, NPs induce stronger toxic responses than do larger-size particles of the same chemical composition (de Haar et al. 2006; Nurkiewicz et al. 2008; Oberdörster et al. 2005). Considering the rapid growth of nanotechnology, it is critical to evaluate the potential health risks associated with NP exposure.

Human exposure to NPs is possible via various routes (i.e., dermal, ingestion, injection for medical purposes), but inhalation is considered the major route of exposure for NPs, especially in occupational settings (Medina et al. 2007; Oberdörster et al. 2005). Therefore, knowledge gained from toxicological studies with ambient nanosized (ultrafine) particles has been used as a valuable database for predicting the toxicology of NPs (Oberdörster et al. 2007). Because there have been reports of associations between inhaled ambient ultrafine particles and increased risk of cardiopulmonary diseases (Pope et al. 2004), it has been suggested that inhaled NPs might have the potential to induce systemic cardiovascular toxicity as well. In support of this notion, a few recent studies have reported the cardiovascular effects of NPs after exposure via the pulmonary route (LeBlanc et al. 2010; Li et al. 2007; Nurkiewicz et al. 2008).

Several mechanisms have been proposed to explain how pulmonary NP exposure can elicit cardiovascular responses. One hypothesis states that NPs deposited in the lung act through a neural mechanism to alter cardiac autonomic function (Nurkiewicz et al. 2006). A second hypothesis proposes that NPs deposited in the lung initiate local inflammatory responses via oxidative stress that further develop into systemic oxidative stress/inflammation (Yamawaki and Iwai 2006). A third hypothesis proposes that NPs deposited in the lung could translocate into the systemic circulation and directly interact with cardiovascular tissues to induce injury or inflammation (Yamawaki and Iwai 2006). Although there is evidence to support all three mechanisms (and they are not mutually exclusive), Brook (2008) suggested that the chronic effects of ambient particles upon the cardiovascular system, such as atherogenesis, were mainly due to the generation of a chronic proinflammatory state.

Recently, our group showed that inhalation of nickel (Ni) hydroxide [Ni(OH)_2_] NPs (nano-NH), a material of growing interest in industry, including power/energy markets (Rocha et al. 2009), could induce significant pulmonary inflammation in mice in a dose-dependent manner (Gillespie et al. 2010). Pulmonary inflammation can lead to systemic inflammation (Yamawaki and Iwai 2006), and given the association between systemic inflammation and various cardiovascular diseases (Sin and Man 2003), it is logical to investigate possible cardiovascular effects of inhaled nano-NH. Furthermore, some evidence from other studies suggests that inhaled Ni can induce systemic cardiovascular effects (Campen et al. 2001; Lippmann et al. 2006). Although the exact mechanisms behind these findings are still unclear, together these data suggest that inhaled nano-NH may induce significant toxicity on the cardiovascular system.

In this study, we investigated whether inhaled nano-NH could induce oxidative stress and inflammatory responses, not only in the lung but also in the extrapulmonary organs, and whether long-term exposure could exacerbate the progression of atherosclerosis in the apoprotein E-deficient (ApoE^−/−^) mouse, a sensitive animal model.

## Materials and Methods

### Animals

We used the ApoE^−/−^ mouse, an animal model widely used to study the development of atherosclerosis. Male ApoE^−/−^ mice were obtained from Taconic Farms (Germantown, NY) and housed in our animal facility, which is accredited by the Association for Assessment and Accreditation of Laboratory Animal Care. Animals were provided with standard rodent chow (TD7001, 4% fat; Harlan Teklad, Indianapolis, IN) and water *ad libitum*. The mice were 5 months of age when exposures began and had at least a 2-week acclimation period before being used for experiments. All procedures involving animals were conducted in compliance with guidelines for ethical animal research and approved by the New York University School of Medicine Animal Care and Use Committee. The animals were treated humanely and with regard for alleviation of suffering.

### Exposure and sacrifice protocol

Details of nano-NH generation and a whole-body exposure system used in this study have been described previously (Gillespie et al. 2010). Animals were exposed either to filtered air (control) or to nano-NH at the nominal concentration of 100 μg Ni/m^3^ for 5 hr/day, 5 day/week, for either 1 week (1w; *n* = 6/group) or 5 months (5m; *n* = 16/group) and sacrificed 24 hr after the last day of exposure with an overdose of sodium pentobarbital via intraperitoneal injection. The average exposure concentration during the study period was 78.6 μg Ni/m^3^ and 79.0 μg Ni/m^3^ for the 1w and 5m studies, respectively [see Supplemental Material, Table 1a (doi:10.1289/ehp.1002508)]. Secondary uptake of nano-NH via ingestion from the material deposited on the exposure chamber or on the animal’s coat was considered negligible, based on preliminary analyses (Gillespie et al. 2010).

### Ni content analyses

The organs of interest (lung, liver, heart, spleen, and whole blood) were removed from each animal, weighed, and wet-ashed in Teflon beakers using Optima-grade nitric acid and hydrogen peroxide (both from Fisher Scientific, Pittsburgh, PA). The samples were analyzed for Ni content using graphite furnace–atomic absorption spectroscopy (GF-AAS; model GF95; Thermo Scientific, Waltham, MA).

### Bronchoalveolar lavage fluid (BALF) analyses

We used BALF to assess recruitment of neutrophils and protein leakage as markers of inflammatory responses and lung damage, respectively. Details of BALF analyses methods have been previously described (Gillespie et al. 2010).

### Real-time reverse-transcriptase polymerase chain reaction (RT-PCR)

To quantify relative mRNA levels of the genes of interest in target tissue, real-time RT-PCR was performed using primer/probe sets from TaqMan Gene Expression Assays and the 7300 Real-Time PCR System (Applied Biosystems, Foster City, CA). Relative expression levels were established using the comparative threshold cycle (C_T_) method outlined in the manufacturer’s instructions for the PCR system. All expression levels were normalized to the housekeeping gene, hypoxanthine phosphoribosyltransferase (*Hprt-1*) and reported as a relative fold change over control; see Supplemental Material (doi:10.1289/ehp.1002508) for additional details.

### Long PCR for assessment of mitochondrial DNA (mtDNA) damage

To measure the relative amount of damaged mtDNA, we used a semiquantitative long PCR assay, conducted according to the protocol of Li et al. (2007), with slight modifications [for details, see Supplemental Material (doi:10.1289/ehp.1002508)].

### Serum analyses

Blood samples were collected by heart puncture, and serum was separated and stored at –80°C until analysis. Serum levels of selected inflammatory markers were determined by the Luminex multiplex system (Luminex Corp., Austin, TX), using commercial kits (BioRad, Hercules, CA). We measured low-density lipoprotein (LDL) and total cholesterol levels using commercial kits (Thermo Scientific).

### Lung histopathology

The left lung lobes from each mouse were drop-fixed in 10% zinc-formalin (Fisher Scientific, Pittsburgh, PA) and then embedded in paraffin. Coronal sections (5 μm) stained with hematoxylin and eosin (H&E) were analyzed by a board-certified pathologist with expertise in thoracic pathology (A.L.M.).

### Plaque area quantification

The aorta was harvested and fixed in 10% zinc-formalin, embedded in paraffin, and cross-sectioned at a 5 μm for H&E staining. We determined the relative plaque area by measuring the size of atherosclerotic lesions over the total aortic cross-sectional area, at four different locations of the ascending aorta separated by 80-μm intervals starting from the aortic root toward the aortic arch. Three consecutive sections were measured for each location.

### Statistical analyses

Differences between the two experimental groups were analyzed using Student’s *t*-test with equal or unequal variance depending on *F*-test results. For comparisons among multiple groups, we used one-way analysis of variance with either Tukey’s or Dunnet’s post hoc test. A *p*-value < 0.05 was considered significant. All statistical analyses were performed using GraphPad Prism software (version 5; GraphPad Software Inc., San Diego, CA).

## Results

### Ni deposition and translocation

The diameter of primary nano-NH particles was approximately 5 nm, and the count median diameter of agglomerates was 40 nm with a geometric SD of 1.50. Particle characteristics of nano-NH have been described previously (Gillespie et al. 2010). The average exposure concentrations of nano-NH were 78.6 μg Ni/m^3^ and 79.0 μg Ni/m^3^ for 1w and 5m groups, respectively [see Supplemental Material, Table 1a (doi:10.1289/ehp.1002508)], and these are < 10% of the current Occupational Safety and Health Administration (OSHA) permissible exposure limit (PEL) for Ni(OH)_2_ (1 mg Ni/m^3^; TOXNET Toxicology Data Network 2003). Following both exposure periods, we assessed the lung Ni burden at 24 hr postexposure. The average amounts of Ni found in the whole lung were 46.9 ng for the 1w group and 306.7 ng for the 5m group (see Supplemental Material, Table 1b). This indicates that continuous exposure to nano-NH caused an accumulation of Ni in the lung over time, despite its relatively rapid clearance from the lung (~ 70% clearance in the first 24 hr), as reported previously (Gillespie et al. 2010). We also analyzed the liver, heart, spleen, and whole blood for Ni content but did not observe significant differences between nano-NH–exposed animals and control animals in any tissue. However, a slight but significant increase (*p* < 0.05) in Ni concentration was observed in the whole blood of wild-type mice (C57BL/6) exposed to nano-NH at a higher concentration (~ 1,000 μg/m^3^) for 3 days [control, 8.17 ± 4.48 ng Ni/mL blood; nano-NH, 24.33 ± 12.86 ng Ni/mL blood (Kang GS, Gillespie PA, Chen L-C, unpublished data).

### Oxidative stress induced by nano-NH exposure

**Figure 1:**
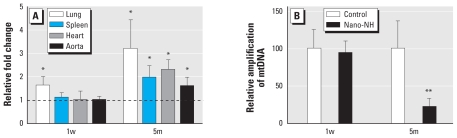
Markers of oxidative stress in mice after 1w or 5m nano-NH exposure (79 μg Ni/m^3^ nano-NH). (*A*) Relative *Ho-1* mRNA levels in various organs; values are mean ± SD (*n* = 6/group) expressed as relative fold increase over controls (normalized to 1; dashed line). (*B*) mtDNA damage in the aorta of ApoE^−/−^ mice, determined by reduction in relative amplification compared with control (100%); values are mean ± SD (*n* = 6/group). Tissue samples were collected 24 hr after the last exposure. *p < 0.05, and **p < 0.01 compared with control.

We determined mRNA levels of selected antioxidant enzymes [nicotinamide adenine dinucleotide phosphate (NADPH) oxidase 1 (*Nox-1*), superoxide dismutase 2 (*Sod-2*), thioredoxin reductase 1 (*Txnrd-1*), and heme oxygenase 1 (*Ho-1*) in lung tissue. Of these genes, only *Ho-1* expression was changed. *Ho-1* was significantly up-regulated in both 1w and 5m exposure groups; the 5m group had a greater fold-increase than did the 1w group (Figure 1A). We consistently found no difference in mRNA abundance of *Nox-1*, *Sod-2*, or *Txnrd-1* in the extrapulmonary organs (spleen, heart, and aorta) from either exposure group (1w and 5m), but *Ho-1* expression was significantly up-regulated in all three organs tested in the 5m group (Figure 1A). When we assessed mtDNA damage as a marker of oxidative stress specifically in the aorta, the results showed dramatically reduced amplification of aortic mtDNA in the 5m nano-NH–exposed mice, which reflects an increase in mtDNA damage, compared with control mice (Figure 1B). All these results suggest that long-term exposure to inhaled nano-NH can induce both pulmonary and systemic oxidative stress in ApoE^−/−^ mice.

### Pulmonary inflammation induced by nano-NH exposure

**Figure 2:**
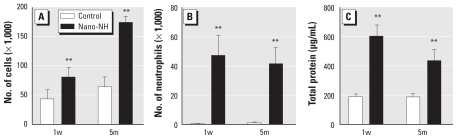
Pulmonary responses measured in BALF in mice after 1w or 5m nano-NH exposure (79 μg Ni/m^3^ nano-NH). (*A*) Total cell counts. (*B*) Number of neutrophils. (*C*) Total protein concentration. All markers were measured 24 hr after the last exposure; values are mean ± SD (*n* = 6/group). **p < 0.01 compared with control.

**Figure 3:**
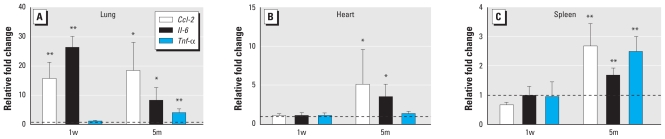
Transcriptional changes of selected proinflammatory genes in mice after 1w or 5m nano-NH exposure (79 μg Ni/m^3^ nano-NH) shown as relative mRNA levels of selected proinflammatory cytokine/chemokine genes in (*A*) lung, (*B*) heart, and (*C*) spleen. All tissue samples were collected 24 hr after the last exposure; values are mean ± SD (*n* = 6/group) expressed as relative fold increase over controls (normalized to 1; dashed line). *p < 0.05, and **p < 0.01 compared with control.

**Figure 4:**
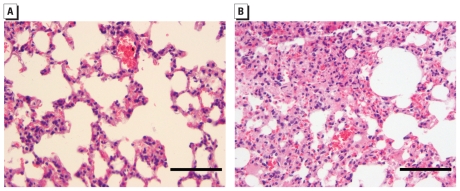
Effects of 5m exposure to nano-NH (79 μg Ni/m^3^ nano-NH shown by histopathological analysis of the mouse lung. Representative H&E-stained images from (*A*) a control mouse showing open alveolar spaces and no inflammatory infiltrate and (*B*) a nano-NH–exposed mouse showing an ill-defined inflammatory nodule composed predominantly of large eosinophilic macrophages infiltrating the pulmonary parenchyma; bars = 0.1 mm. Tissue samples were collected 24 hr after the last exposure.

In BALF from both 1w and 5m exposure groups, we found significant increases in total cell counts, neutrophil influx, and protein leakage (Figure 2), which indicate persistent inflammation and tissue injury induced by nano-NH exposure. For transcriptional changes in the lung tissue, we assessed relative mRNA abundance of selected proinflammatory cytokines/chemokines: chemokine (C-C motif) ligand 2 (*Ccl-2*), interleukin-6 (*Il-6*), and tumor necrosis factor-α (*Tnf-*α). In the 1w exposure group, only *Ccl-2* and *Il-6* showed significantly increased mRNA levels, whereas all three genes were up-regulated in the 5m exposure group (Figure 3A). The histopathological analysis also revealed distinct differences in the lung tissue between control and nano-NH–exposed mice in the 5m group. We observed mild to moderate inflammatory infiltrate composed predominantly of large eosinophilic macrophages and lymphocytes in the lung of nano-NH–exposed mice (Figure 4B). The inflammatory infiltrate was ill-defined and could be found in the peribronchial and interstitial area of the lung. In addition, focal bronchiolitis was also present. In contrast, the lungs of control mice appeared normal with no significant inflammatory infiltrate (Figure 4A). These results are consistent in indicating persistent pulmonary inflammation induced by nano-NH exposure.

### Systemic inflammation induced by nano-NH exposure

Serum amyloid P component (SAP) is a major acute-phase protein in the mouse that is produced mainly by Kupffer cells in the liver following injury or inflammation. Thus, we assessed the abundance of *Sap* mRNA in the livers of mice as a sensitive marker of systemic inflammation and found significantly increased levels after exposure to nano-NH in both the 1w group (3.5-fold) and the 5m group (3.6-fold) [see Supplemental Material, Figure 1 (doi:10.1289/ehp.1002508)]. Other markers of systemic inflammation used in this study were the mRNA levels of proinflammatory cytokines and chemokines in the heart and spleen. We measured relative mRNA levels of *Ccl-2*, *Il-6*, and *Tnf-*α in each organ (Figure 3B,C). In the 1w exposure group, we observed no significant transcriptional changes in either organ. However, in the 5m exposure group, *Ccl-2* and *Il-6* were up-regulated in the heart, and all three genes were up-regulated in the spleen. These findings suggest that prolonged exposure to inhaled nano-NH can induce systemic inflammatory responses in extrapulmonary organs such as the heart and spleen, although no significant difference was observed in serum levels of those cytokines/chemokines between control and nano-NH groups.

### Progression of atherosclerosis

**Figure 5:**
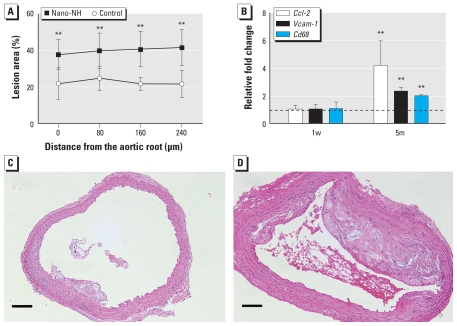
Effects of nano-NH exposure (79 μg Ni/m^3^ nano-NH) on progression of atherosclerosis. (*A*) Relative plaque area at four different locations of the ascending aorta (at 80-μm intervals) in the 5m group; values are expressed as mean ± SD (*n* = 7/group). (*B*) Relative mRNA levels of *Ccl-2*, *Vcam-1*, and *Cd68* in aortas from the 1w and 5m exposure groups; values are mean ± SD (*n* = 6/group) expressed as relative fold increase over controls (normalized to 1; dashed line). (*C* and *D*) Photomicrographs of representative H&E-stained aortic cross-sections from control (*C*) and nano-NH (*D*) mice in the 5m group; bars = 0.2 mm. All tissue samples were collected 24 hr after the last exposure. **p < 0.01 compared with control.

To determine the relative degree of progression of atherosclerosis, we measured relative plaque area using cross-sectional slides of the ascending aorta. At all four locations of the ascending aorta (with 80-μm intervals from the aortic root), mice in the 5m nano-NH exposure group had significantly greater lesion area than did control mice (Figure 5A,C,D). We found no difference in plaque size among locations. Overall, plaque formation in the ascending aorta of the 5m exposure group was approximately 1.8 times that of the controls (Figure 5A). In addition, we selected three genes involved in atherogenesis, *Ccl-2*, vascular cell adhesion molecule 1 (*Vcam-1*), and cluster of differentiation 68 (*Cd68*), and determined their mRNA levels in thoracic aorta tissue. mRNA levels of all three genes increased significantly in the 5m exposure group (Figure 5B). To test whether nano-NH exposure affected lipid metabolism in mice, we measured serum levels of LDL and total cholesterol. Neither marker showed significant differences between nano-NH and control groups after both exposure periods (data not shown).

## Discussion

To the best of our knowledge, this is the first study to investigate long-term cardiovascular toxicity of an inhaled nanomaterial. Our results clearly demonstrate that subchronic inhalation exposure to nano-NH can induce significant cardiovascular effects including exacerbation of atherosclerosis in ApoE^−/−^ mice.

After exposure to inhaled nano-NH, ApoE^−/−^ mice showed signs of both pulmonary and extrapulmonary oxidative stress, such as marked transcriptional up-regulation of *Ho-1* in the lungs and other tissues (spleen, heart, and aorta) and mtDNA damage in the aorta. We chose to measure *Ho-1* expression because it has been widely used as a sensitive marker for oxidative stress, especially in particle-mediated toxicity studies (Eom and Choi 2009; Lund et al. 2007; McDonald et al. 2010). *Ho-1* is transcriptionally up-regulated by various types of oxidative stress (Anwar et al. 2005) and is highly expressed in atherosclerotic lesions (Ishikawa 2003). Our results clearly show that exposure to inhaled nano-NH increased mRNA levels of *Ho-1* in the lung in both 1w and 5m exposure groups and also in the heart, spleen, and aorta in the 5m exposure group. Up-regulation of *Ho-1* in the aorta was especially interesting because it coincided with increased aortic mtDNA damage. It has been suggested that *Ho-1* inhibits oxidized LDL-dependent monocyte chemotaxis, and therefore, *Ho-1* induction can act as a protective mechanism against vascular injuries (Ishikawa 2003). On the other hand, mtDNA is known to be highly susceptible to oxidative stress, and the role of mtDNA damage in various human diseases related to oxidative stress, including atherosclerosis, has been widely studied (Ballinger 2005). mtDNA damage in both human and mouse vascular tissues has been correlated with the extent of atherosclerosis (Puddu et al. 2005), and it has also been suggested to be an initiating event in atherogenesis (Ballinger et al. 2002). Therefore, the fact that there was significant mtDNA damage in the aorta in the 5m nano-NH exposure group despite up-regulated *Ho-1* suggests that oxidative stress produced by inhaled nano-NH overwhelmed the capacity of *Ho-1*–related defense mechanisms to fully compensate for the nano-NH–induced oxidative stress.

We have also shown that pulmonary and systemic inflammation is induced by inhaled nano-NH. To assess pulmonary inflammation, we used commonly studied markers such as neutrophil influx and protein leakage in BALF, mRNA levels of major inflammatory cytokines and chemokines, and histological analyses. The results of all of these analyses indicate that significant and persistent pulmonary inflammation was caused by inhaled nano-NH. Further, we found that some of the same inflammatory cytokine and chemokine genes that were up-regulated in the lung were also up-regulated in the heart and spleen, suggesting systemic inflammation. One of the interesting end points used in this study to measure systemic inflammation was hepatic mRNA levels of a mouse acute-phase protein, *Sap*. Although C-reactive protein, a major acute-phase protein in humans, has been widely studied as a biomarker for cardiovascular disease in humans, few studies have investigated acute-phase responses in the mouse. Because SAP is a major acute-phase protein in the mouse (Saber et al. 2009; Whitehead et al. 1990), we assessed the level of *Sap* mRNA in the liver as a sensitive marker for systemic inflammation. In both exposure groups (1w and 5m), *Sap* mRNA levels in the liver increased significantly compared with controls, further suggesting the induction of systemic inflammation by inhaled nano-NH.

To determine the effects of inhaled nano-NH on development of atherosclerosis in ApoE^−/−^ mice, we quantified plaque formation on the ascending aorta. We chose the ascending aorta as the site of measurement because it is known to have a predilection for development of atherosclerosis in ApoE^−/−^ mice (Nakashima et al. 1994), and the plaque area of this site is closely correlated to total plaque burden of the entire aorta (Gan et al. 2007). At all four locations selected on the ascending aorta, the mice in the 5m nano-NH exposure group had significantly larger plaques (~ 1.8-fold) than the control mice. We sought to identify characteristics of the plaques that might provide insights to mechanisms that could explain the acceleration of plaque formation in nano-NH–exposed mice. Hence, we measured the abundance of *Ccl-2*, *Vcam-1*, and *Cd68* transcripts in the aorta. *Ccl-2* is well known for its involvement in monocyte adhesion during the early development of atherosclerosis (Kusano et al. 2004). *Vcam-1* is an adhesion molecule that is expressed on activated endothelial cells and known to promote monocyte adhesion and accumulation on the vessel wall in the initiation stage of atherosclerosis (Ma et al. 2008). *Cd68* is a macrophage-specific marker (Graebe et al. 2009) and has been widely used as an indicator of macrophage infiltration into the endothelium during atherogenesis (Moreno et al. 2000; Tearney et al. 2003). We found that all three genes were up-regulated in the 5m nano-NH exposure group. These results provide plausible molecular mechanisms for the increase in atherosclerotic lesion area in mice exposed to nano-NH for 5 months.

Another end point we measured because of its association with atherosclerosis was serum cholesterol. Lipid is one of the most studied and most important stimuli initiating atherogenesis (Mehra et al. 2005), and extracellular accumulation of lipids occurs early in response to increased plasma lipoprotein levels in animals (Skalen et al. 2002). Here we found no significant differences in serum LDL or total cholesterol levels between nano-NH–exposed and control mice, which suggests that the increase in plaque formation after nano-NH exposure might be due to other mechanisms contributing to atherogenesis, such as systemic oxidative stress and/or inflammation.

Although many epidemiological and experimental studies have shown the association between inhaled ambient particles and cardiovascular effects, the mechanistic basis of this association remains largely unknown. Brook (2008) proposed generation of a chronic proinflammatory state as a major mechanism for chronic effects of ambient particles upon the cardiovascular system. A key question regarding this potential mechanism is whether a systemic proinflammatory state is induced by mediators secreted from the exposed lung or the result of direct interaction between translocated particles and local tissues.

In a recent study, Mitchell et al. (2009) suggested that tumor growth factor-β released from the lung of mice after inhalation exposure to multiwalled carbon nanotubes could induce the cyclooxygenase pathway in the spleen, leading to systemic immune responses. Their finding was especially interesting because the mice did not show any overt lung inflammation. Considering the signs of significant pulmonary inflammation in nano-NH–exposed mice, it is possible that certain mediators released from the inflamed lung could have induced further systemic effects in the present study as well. However, we found that most markers for systemic effects showed significant changes only in the 5m group, not in the 1w group, whereas signs of strong pulmonary inflammation were evident in the 1w exposure group. This suggests that some of the observed systemic effects (i.e., increased mRNA levels in extrapulmonary tissues) may be due to the persistent interaction of small quantities of translocated nano-NH with target tissues over time. Although we found no significant differences in Ni content between control and nano-NH–exposed groups in the heart, liver, or spleen, this is not surprising considering that *a*) our exposure concentration was fairly low and *b*) Ni had relatively high baseline levels and large individual variation, probably due to its relatively high content in rodent chow (2–3 μg Ni/g chow). Furthermore, we observed a slight but statistically significant increase in Ni concentration in the blood after a 3-day exposure to a higher concentration of nano-NH (~ 1,000 μg/m^3^) in wild-type C57BL/6 mice (Kang GS, Gillespie PA, Chen L-C, unpublished data). This may be an indication of translocation, supporting the hypothesis of adverse systemic effects caused by translocation of NPs. In a recent study, Wallenborn et al. (2007) showed that ambient particulate matter–associated metals (including Ni) were rapidly translocated to the circulation after intratracheal instillation, and this translocation was dependent on the solubility of metals. Given the high dissolution rate of nano-NH [~ 86% within 24 hr in physiological buffer (Gillespie et al. 2010)], it is possible that a fraction of inhaled nano-NH was translocated to systemic circulation and accumulated in extrapulmonary organs over time. Future studies using Ni-deficient chow and more sensitive analytical methods to detect minute changes in serum inflammatory markers, as well as Ni translocation, may assist in further elucidating the mechanisms of systemic effects caused by inhaled nano-NH.

One of the major strengths of the present study is that by using a whole-body inhalation system and an occupationally realistic exposure concentration, the exposure conditions were relevant to human exposure scenarios. Inhalation exposure is considered the gold standard for assessing the toxicity of airborne materials (Elder 2009), and in our previous study (Gillespie et al. 2010), we described key advantages of using a whole-body exposure system. In addition, we used an exposure concentration that is < 10% of the current occupational guideline (OSHA PEL) for Ni(OH)_2_. Although no actual workplace exposure data have yet been reported for nano-NH, 79 μg Ni/m^3^ is relatively low compared with the occupational exposure concentrations in other industries using Ni (Sivulka et al. 2007).

## Conclusion

The results of our study clearly demonstrate that inhaled nano-NH induced significant oxidative stress and inflammation in the pulmonary and extrapulmonary organs. In addition, over the long-term (5 months), nano-NH exacerbated the progression of atherosclerosis in a sensitive mouse model. We believe that these findings will contribute to the further understanding of potential risks and mechanisms of NP-induced toxicity and to establishing a database for NP-specific regulations in occupational settings.
